# DA-HGL: a domain-augmented heterogeneous graph learning framework for protein function prediction

**DOI:** 10.1093/bib/bbaf511

**Published:** 2025-09-28

**Authors:** Sai Hu, Wei Zhang, Bihai Zhao

**Affiliations:** School of Mathematics, Changsha University, No. 98 Hongshan Road, Changsha, Hunan 410022, China; School of Computer Science and Engineering, Changsha University, No. 98 Hongshan Road, Changsha, Hunan 410022, China; Hunan Provincial Key Laboratory of Industrial Internet Technology and Security, Changsha University, No. 98 Hongshan Road, Changsha, Hunan 410022, China; School of Computer Science and Engineering, Changsha University, No. 98 Hongshan Road, Changsha, Hunan 410022, China; Hunan Provincial Key Laboratory of Industrial Internet Technology and Security, Changsha University, No. 98 Hongshan Road, Changsha, Hunan 410022, China

**Keywords:** protein function prediction, heterogeneous graph learning, Gene Ontology, domain architecture, disease mechanism

## Abstract

Accurate protein function prediction is critical for deciphering disease mechanisms and advancing precision medicine, yet remains challenging for proteins with sparse annotations. Traditional methods struggle with annotation sparsity and fail to integrate multimodal data holistically. We propose DA-HGL, a heterogeneous graph learning framework that integrates protein sequences, domain architectures, and Gene Ontology (GO) hierarchies through a multilayered graph and non-negative matrix factorization with dual biological constraints. DA-HGL uniquely models domain-function coherence, GO semantic consistency, and topological congruence. Evaluated on yeast and human proteomes, DA-HGL achieves *F_max_* gains of 9.0% (yeast CC) and 17.2% (human BP) over state-of-the-art methods. By dynamically learning domain-context associations and resolving annotation sparsity, DA-HGL excels in cold-start scenarios and disease-specific predictions (e.g. Parkinson’s “ubiquitin-dependent catabolism”). This framework offers a robust tool for accelerating functional genomics and precision medicine. Code/data: https://github.com/husaiccsu/DA-HGL.

## Introduction

Proteins are fundamental executors of cellular processes, and their functional dysregulation underpins numerous diseases [1], from metabolic disorders to neurodegenerative conditions. Accurate functional annotation of proteins is therefore pivotal not only for deciphering disease mechanisms [2] but also for accelerating drug discovery in precision medicine [3]. Early methods mainly relied on protein–protein interaction (PPI) networks, predicting functions based on the “guilt-by-association” principle that interacting proteins often share functions [4]. These methods inferred functions via network topological features but had low reliability due to the high false positive rates (30%–60% in yeast and human data) [5] and low coverage of PPI data, especially for uncharacterized proteins. Subsequently, researchers integrated multimodal data. Sequence based deep learning methods (such as DeepGOMeta [6] and protein language models [7]) quickly predicted functions by capturing evolutionary conservation but ignored the contextual associations between proteins and their interaction partners. Domain-based methods (like DSCP [8], Domain-PFP [9], and GrAPFI [10]) leveraged the conservation of functional modules but failed to model the hierarchical semantics of Gene Ontology (GO) and the dependencies across multimodal data (e.g. sequences and interaction networks).

The GO organizes functional terms in a directed acyclic graph (DAG), where child terms (e.g. “tyrosine kinase activity”) inherit hierarchical semantics from parents (e.g. “kinase activity”). Existing methods (exp2GO [11], GO embeddings [12]), relying on single data types, struggle with annotation sparsity for context-dependent functions. While recent sequence-based deep learning methods (e.g. DeepPFP [13]) leverage protein language models for function prediction, they remain limited by omitting domain architecture contexts critical for tissue-specific functions and ignoring GO semantic hierarchies that enforce biological consistency. To address these challenges, we propose a novel framework, DA-HGL (Domain-Augmented Heterogeneous Graph Learning), which integrates protein domain architectures, sequence evolutionary features, and GO hierarchical constraints to construct a multilayer heterogeneous graph model based on our prior work in heterogeneous network analysis [14]. The framework enforces three biological constraints through non-negative matrix factorization (NMF). Specifically, it ensures domain-function consistency by assuming proteins sharing domains exhibit similar functional spectra. Additionally, it maintains GO semantic consistency by enforcing predicted functions to adhere to ontological hierarchies, such as requiring child terms to inherit parent term annotations. Furthermore, the framework enforces topological homogeneity, where functionally related proteins form tight connections in the heterogeneous graph via shared domains, GO terms, or other intermediaries. The main contributions of this work can be summarized as follows:

(1) Dynamic domain embeddings: Context-aware representations via sequence-GO co-optimization, enabling tissue-specific predictions.
(2) Hierarchical semantic propagation: A GO-GO graph with weighted “is_a”/“part_of” edges to resolve inconsistencies.
(3) Cold-start mitigation: Alignment-free sequence features (PseAAC [15]) combined with domain-augmented similarities.
(4) Biomedical relevance: Identifies disease-specific terms (e.g. Parkinson’s “ubiquitin-dependent catabolism” [16]), aiding therapeutic target discovery.

## Materials and methods

**Figure 1:**
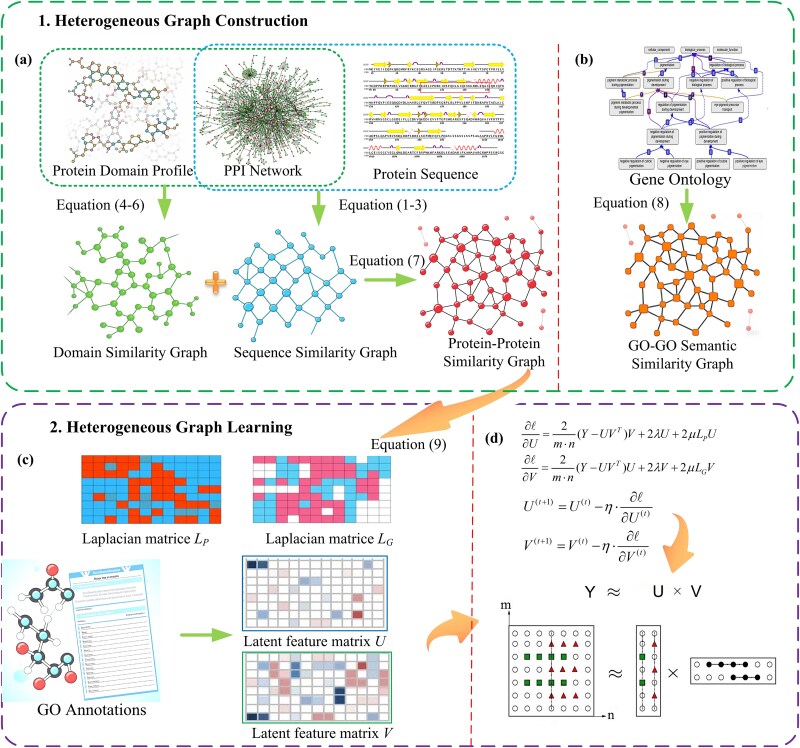
The flowchart of the DA-HGL method. The DA-HGL framework comprises two sequential phases: heterogeneous graph construction and heterogeneous graph learning. (a) Protein–protein graph: Combines sequence (AAC/PseAAC) and domain (TF-IDF) similarities. (b) GO semantic graph: Models “is_a”/“part_of” hierarchies via shortest-path aggregation. (c) Dual regularization: Laplacians (*L_P_*, *L_G_*) with SVD-initialized embeddings. (d) Optimization: Gradient descent minimizes reconstruction error, outputting protein-GO predictions.

Our framework illustrated in Fig. 1 operates in two sequential phases: heterogeneous graph construction and graph-guided functional inference. First, we integrate multimodal data, such as protein sequences, domain architectures, and GO annotations to construct a heterogeneous graph with three interaction layers. Next, we formulate a NMF model with dual graph regularization, enforcing domain-function coherence and GO semantic consistency. By jointly optimizing latent protein and GO term embeddings through gradient descent, our model predicts functional annotations while preserving biological principles of topological congruence and hierarchical inheritance.

### Heterogeneous graph construction

A heterogeneous graph is defined as *G* = (*V*, *E*), where $V={V}_P\cup{V}_G$ contains two types of nodes: protein nodes (*V_P_*) and GO term nodes (*V_G_*). Three edge types are incorporated to model multiscale relationships: Protein–protein similarity edges (*E_PP_*), Protein-GO association edges (*E_PG_*), and GO-GO semantic similarity edges (*E_GG_*). This framework aligns with recent advances in systems biology, where heterogeneous graphs have proven effective for tasks like drug-target prediction [17] and disease gene prioritization [18]. Below we detail the construction of each subgraph.

Traditional alignment-based methods may not capture subtle similarities in nonhomologous sequences. In this study, we explore two alignment-free methods: Amino Acid Composition (AAC) and Pseudo Amino Acid Composition (PseAAC). The AAC vector of sequence *s* is defined as:


(1) \begin{equation*} {V}_{AAC}\left[i\right]=\frac{count\left(A\left[i\right],s\right)}{\mid s\mid },\kern0.75em i=1,2,\dots, 20 \end{equation*}

Where *count* (*A*[*i*], *s*) is the number of occurrences of the *i*-th amino acid in sequence *s*, and |*s*| is the length of sequence *s*. The PseAAC vector is an extension of the AAC vector, which takes into account the sequence order information. Given a sequence *S* and parameters λ and *w*, the PseAAC vector is calculated as:


(2) \begin{equation*} {V}_{PseAAC}={V}_{AAC}\cup{\left[w\cdot corr\left(s,l\right)\right]}_{l=1}^{\lambda } \end{equation*}

Where λ denotes the maximum lag value (default: 20), *w* is the weighting factor (default: 0.05), *corr*(*s*, *l*) represents the autocorrelation function at lag *l* (ranging from 1 to λ) and is defined as follow:


(3) \begin{equation*} corr\left(s,l\right)=\frac{1}{\mid s\mid -l}\sum \limits_{i=1}^{\mid s\mid -l}\left\{\!\!\begin{array}{l}1\kern2.25em if\ A\left[i\right]=A\left[i+l\right]\kern1em \\{}-1\kern4.5em otherwise\end{array}\right. \end{equation*}

The similarity $Si{m}_{seq}\left({s}_1,{s}_2\right)$ between two protein sequences *s*_1_ and *s*_2_ is calculated using the cosine similarity of their PseAAC vectors. Therefore, the sequence-based similarity network is constructed by calculating the similarity between protein pairs with experimentally validated interactions in the PPI network, reducing noise from unrelated proteins.

For domain information, we propose a hybrid TF-IDF approach considering both intrinsic domains and neighbor associated domains. For protein *i*, we collect its native domains *D_i_* and compute TF-IDF weights for each domain *d*∈ *D_i_*:


(4) \begin{equation*} {w}_d^{(i)}= tf\left(d,{D}_i\right)\cdot \log \left(\frac{N}{n_d+1}\right) \end{equation*}

Where *tf*(*d*,*D_i_*) denotes the binary term frequency, *tf*(*d*,*D_i_*) = 1 if domain *d* exists in *D_i_*, otherwise 0. *N* is total number of proteins in the dataset, and *n_d_* is number of proteins containing domain *d*. To capture functional contexts from protein domain profile, we aggregate domains from both protein *i* and its direct neighbors *N_i_* in the PPI network.


(5) \begin{equation*} {D}_i^{aug}={D}_i\cup \left(\underset{k\in{N}_i}{\cup }{D}_k\right) \end{equation*}

The final similarity between proteins *i* and *j* combines their native and augmented domain representations using a weighted cosine similarity:


(6) \begin{equation*} Si{m}_{Domain}\left(i,j\right)=\beta \cdot \cos \left({w}_i,{w}_j\right)+\left(1-\beta \right)\cdot \cos \left({w_i}_{aug},{w_j}_{aug}\right) \end{equation*}

Where *w_i_* and ${w_i}_{aug}$ denotes the TF-IDF vector of native domains *D_i_* and augmented domains ${D}_i^{aug}$ using Equation (4), respectively. The weight parameter β prioritizing neighbor augmented contexts is set to 0.1 [8]. The protein–protein similarity graph combines sequence and domain similarities through species-specific weighting:


(7) \begin{equation*} {E}_{PP}\left(i,j\right)=\gamma \times Si{m}_{seq}+\left(1-\gamma \right)\times Si{m}_{Domain} \end{equation*}

In the protein-GO association graph, the association between proteins and GO terms is directly curated from the Gene Ontology Annotation (GOA) database, where edges represent experimentally validated annotations. In the protein-GO association graph, a binary adjacency indicator *E_PG_*(*i,j*) is established: when protein *p_i_* is experimentally annotated with GO term *g_j_*, an edge is drawn between the corresponding nodes in the bipartite graph, setting *E_PG_*(*i,j*) = 1; conversely, the absence of an annotation relationship is denoted by *E_PG_*(*i,j*) = 0. Proteins lacking functional characterization induce sparsity in the *E_PG_* matrix, manifested as missing entries in rows corresponding to unannotated proteins. To address these epistemic gaps, we implement a NMF framework, where imputed elements represent probabilistic annotations that reflect the likelihood of a protein associated with a specific GO term.

To mitigate noise from distant hierarchical relationships, we focus on direct semantic links (immediate parent–child pairs), as empirical analysis shows this strategy enhances the robustness of similarity calculations compared to full-hierarchy approaches. For two GO terms *g_i_* and *g_j_*, their semantic similarity is computed by aggregating weights along the shortest semantic path connecting them:


(8) \begin{equation*} {E}_{GG}\left({g}_i,{g}_j\right)=\sum{}_{\left(u,v\right)\in path\left({g}_i,{g}_j\right)}w\left(u,v\right) \end{equation*}

Where *path*(*g_i_*, *g_j_*) denotes the shortest path in the GO DAG, and *w*(*u*, *v*) assigns edge-specific semantic weights. For “is_a” relationships, the weight *w*(*u*, *v*) is set to 0.4 [19], reflecting strong functional inheritance (e.g. “kinase activity” is a subtype of “tyrosine kinase activity”). In contrast, for “part_of” relationships, the weight *w*(*u*, *v*) is assigned a value of 0.3 [19], capturing structural or compositional associations (e.g. “mitochondrion” is a component of “mitochondrial matrix”). Sensitivity analysis confirmed performance invariance to “is_a” and “part_of” weight variations under the recommended learning rate (η = 0.001).

By restricting the similarity computation to immediate semantic neighbors, the model avoids overcomplicating the graph with indirect relationships while preserving the essential functional hierarchy. The resulting *E_GG_* serves as a critical component in the heterogeneous graph, enabling context-aware propagation of GO term similarities during the function prediction process.

### Heterogeneous graph learning

We propose a heterogeneous graph learning framework that integrates the protein-GO association matrix via NMF, incorporating Laplacian regularization from both the protein similarity graph and the GO semantic graph to achieve functional prediction under domain-function coherence and semantic hierarchy constraints. Given the adjacency matrix of the protein-GO association graph, denoted as $Y\in{R}^{n\times m}$, where *n* and *m* represent the number of proteins and GO terms, respectively, we aim to factorize *Y* into two low-rank non-negative matrices *Y* ≈ *UV^T^*. *U* represents the basis matrix capturing the latent features of proteins and *V* denotes the coefficient matrix indicating the association of GO terms with these latent features, thereby facilitating the analysis and understanding of protein-function relationships.

To initialize the latent feature matrices *U* and *V*, we perform truncated singular value decomposition (SVD) on the full protein-GO association matrix. Specifically, the SVD of the full matrix *Y* is expressed as *Y*=*P*Σ*Q*, where *P* and *Q* are the left and right singular vector matrices, respectively, and Σ is the diagonal matrix of singular values. To prevent information leakage, the rows of *Y* corresponding to test set proteins are set to zero during training (i.e. their functional annotations are treated as unknown), yielding a processed matrix *Y*′. Subsequently, we retain the top *k* largest singular values and their corresponding singular vectors from *Y*′, obtaining the truncated approximation *Y_k_* = *P_k_*Σ*_k_Q_k_*. Here, *P_k_* and *Q_k_* are the truncated left and right singular vector matrices, respectively, while Σ*_k_* is the diagonal matrix containing the top *k* singular values. Finally, *P_k_* and *Q_k_* are used as the initial values for the latent feature matrices *U* and *V*.

To incorporate topological constraints from the heterogeneous graph, we construct Laplacian matrices *L_P_* and *L_G_* for the protein–protein similarity graph and the GO semantic similarity graph, respectively.


(9) \begin{equation*} {L}_P={D}_P-{M}_{PP},\kern0.5em {L}_G={D}_G-{M}_{GG} \end{equation*}

Where *D_P_* and *D_G_* are diagonal degree matrices derived from *E_PP_* and *E_GG_*, respectively. The proposed heterogeneous graph learning framework is formalized as a NMF problem, integrating three key biological constraints into the objective function. The objective function is composed of four components: a reconstruction error term, an *L*2 regularization term, and two graph Laplacian regularization terms for protein–protein similarity and GO semantic similarity, respectively. The complete objective function is defined as:


(10) \begin{equation*} \tau ={\tau}_{recon}+{\tau}_{L2}+{\tau}_P+{\tau}_G \end{equation*}

(11) \begin{equation*} {\tau}_{recon}=\frac{1}{n\cdot m}{\left\Vert Y-U{V}^T\right\Vert}_F^2 \end{equation*}

(12) \begin{equation*} {\tau}_{L2}=\lambda \left({\left\Vert U\right\Vert}_F^2+{\left\Vert V\right\Vert}_F^2\right) \end{equation*}

(13) \begin{equation*} {\tau}_P=\mu \cdot tr\left({U}^T{L}_PU\right) \end{equation*}

(14) \begin{equation*} {\tau}_G=\mu \cdot tr\left({V}^T{L}_GV\right) \end{equation*}

The Frobenius norm-based reconstruction error term τ*_recon_* measures the discrepancy between the observed protein-GO associations and the product of the latent feature matrices *U* (protein embedding) and *V* (GO term embedding). The normalization by *n*.*m* ensures scale invariance across datasets of different sizes and stabilizes gradient magnitudes during optimization. The *L*2 regularization term τ_*L*2_ is applied to prevent over fitting and promote model generalization. λ > 0 is the regularization coefficient controlling the strength of the penalty on large feature values.

The protein similarity graph regularization term τ*_P_* enforces topological consistency in the protein latent space by leveraging the protein–protein similarity graph *E_PP_*. Here, μ is the weight coefficient balancing the influence of the protein similarity structure. This term ensures that proteins close in the similarity graph have similar latent representations, aligning with the “guilt-by-association” principle. The GO semantic similarity graph regularization term τ*_G_* is applied to preserve the hierarchical semantics of GO terms. Notably, *L_P_* and *L_G_* share the same weight coefficient μ, a design choice supported by foundational work in multiview graph learning [20], where balanced regularization ensures proportional contributions from heterogeneous data sources. In this work, μ = 0.1 follows empirical guidelines from domain-specific hyper parameter tuning in biological network analysis [21].

The objective function expressed by Equation (10) is optimized using gradient descent to iteratively update *U* and *V*. The partial derivatives (gradients) of the objective function with respect to *U* and *V* are derived as follows:


(15) \begin{equation*} {\displaystyle \begin{array}{l}\frac{\partial \ell }{\partial U}=\displaystyle\frac{2}{m\cdot n}\left(Y-U{V}^T\right)V+2\lambda U+2\mu{L}_PU\\[6pt] {}\frac{\partial \ell }{\partial V}=\displaystyle\frac{2}{m\cdot n}\left(Y-U{V}^T\right)U+2\lambda V+2\mu{L}_GV\end{array}} \end{equation*}

The latent matrices are updated in the direction of the negative gradient with a learning rate η = 0.001, a value commonly used in matrix factorization-based bioinformatics models to balance convergence speed and stability [22]. The update rules are:


(16) \begin{equation*} {\displaystyle \begin{array}{l}{U}^{\left(t+1\right)}={U}^{(t)}-\eta \cdot \displaystyle\frac{\partial \ell }{\partial{U}^{(t)}}\\[6pt] {}{V}^{\left(t+1\right)}={V}^{(t)}-\eta \cdot \displaystyle\frac{\partial \ell }{\partial{V}^{(t)}}\end{array}} \end{equation*}

The optimization process terminates when the change in the objective function falls below a tolerance threshold 10^−6^, ensuring convergence to a locally optimal solution.

## Results

### Experimental data

We evaluated our proposed method, DA-HGL, alongside eight state-of-the-art protein function prediction methods: Zhang [23], exp2GO [11], GrAPFI [10], PHN [14], DSCP [8], NC [4], Domain-PFP [9], and DeepPFP [13]. We evaluated DA-HGL on two species: yeast (*Saccharomyces cerevisiae*) and human (*Homo sapiens*). PPI networks were filtered to retain interactions supported by at least 3 distinct experimental methods in BioGRID [24], reducing false positives as demonstrated in [5]. (Yeast: 3162 proteins/21,997 interactions; human: 7317 proteins/25,410 interactions). Proteins are named according to the UniProt Knowledgebase (UniProtKB) format [25].

The GO [26] data used for constructing the GO-GO semantic similarity graph were version 1.2. The protein-domain association data was downloaded from the Pfam28 database, version 37.0. We utilized the PFAM-A classification owing to its superior quality and accuracy. The protein sequence data for constructing the protein–protein similarity graph was retrieved from the National Center for Biotechnology Information (NCBI) [27]. To avoid GO terms that are too special or too general [8, 23], we keep only those that annotate at least 10 and at most 200 proteins in the third Critical Assessment of Protein Function Annotation (CAFA3) [28] dataset. Dataset statistics including sequence coverage, domain annotation completeness, and experimental GO term counts are comprehensively detailed in Table 1. The high coverage rates ensure minimal data-driven bias in our evaluation.

**Table 1:** Dataset statistics for yeast and human proteomes.

Data category	Metric	Yeast	Human
Full PPI network	Total proteins	3167	7317
	Proteins with sequences	3095 (97.7%)	6458 (88.3%)
	Proteins with domains	2, 875 (90.8%)	7083 (96.8%)
CAFA3 evaluation set	Proteins with GO terms	2840	5801
	Proteins with sequences	2840 (100%)	5801 (100%)
	Proteins with domains	2602 (91.6%)	5660 (97.6%)

### Evaluation criteria

We adopted CAFA challenge guidelines for evaluation, using leave-one-out cross validation (LOOCV) and 10-fold cross validation (10-CV) on the CAFA3 benchmark. Three metrics were used: *F_max_*, AUPR (area under the precision-recall curve), and AUC (area under the receiver operating characteristic curve). *F_max_* is a threshold-independent metric that balances precision and recall by computing their harmonic mean at the optimal threshold. For a set of predicted probabilities, *F_max_* is defined as:


(17) \begin{equation*} {F}_{\mathrm{max}}=\underset{t}{\max}\left(\frac{2\times precision(t)\times recall(t)}{precision(t)+ recall(t)}\right) \end{equation*}

Where precision(t) and recall(t) denote precision and recall at threshold *t*, respectively. *F_max_* is particularly suited for hierarchical ontologies, as it evaluates the ability of models to maximize both specificity and sensitivity across varying confidence levels.

Statistical significance was assessed via paired t-tests and Wilcoxon signed-rank tests, with FDR correction for multiple comparisons (*P* < 0.05). Results were reported for molecular function (MF), biological process (BP), and cellular component (CC) ontologies separately.

### Leave-one-out cross validation

LOOCV was used to simulate predictions for understudied proteins, with each protein sequentially held out as the test set. We compared DA-HGL against two representative baseline methods: Zhang and Domain-PFP. The results, summarized in Table 2, demonstrate superior performance of DA-HGL across all evaluation metrics and ontology categories.

**Table 2:** Evaluation of different methods using leave-one-out cross validation

Categories	Methods	Yeast			Human		
		*F_max_*	AUC	AUPR	*F_max_*	AUC	AUPR
BP	zhang	0.211	0.874	0.127	0.162	0.798	0.09
	DSCP	0.284	0.915	0.187	0.222	0.861	0.147
	NC	0.359	0.888	0.261	0.3	0.766	0.204
	PHN	0.464	0.924	0.399	0.318	0.832	0.213
	**DA-HGL**	**0.515**	**0.939**	**0.498**	**0.384**	**0.872**	**0.333**
	exp2GO	0.447	0.865	0.378	0.322	0.756	0.236
	GrAPFI	0.174	0.585	0.049	0.1	0.661	0.023
	Domain-PFP	0.494	0.868	0.399	0.31	0.825	0.227
	DeepPFP	0.377	0.861	0.287	0.288	0.822	0.203
MF	zhang	0.203	0.78	0.119	0.115	0.743	0.051
	DSCP	0.355	0.869	0.233	0.228	0.851	0.136
	NC	0.291	0.778	0.187	0.168	0.7	0.075
	PHN	0.379	0.834	0.308	0.194	0.792	0.107
	**DA-HGL**	**0.533**	**0.897**	**0.519**	**0.379**	**0.878**	**0.302**
	exp2GO	0.419	0.751	0.316	0.238	0.681	0.125
	GrAPFI	0.374	0.655	0.215	0.274	0.708	0.106
	Domain-PFP	0.437	0.881	0.377	0.366	0.906	0.265
	DeepPFP	0.490	0.876	0.460	0.320	0.838	0.226
CC	zhang	0.281	0.847	0.205	0.163	0.757	0.087
	DSCP	0.347	0.904	0.275	0.252	0.839	0.15
	NC	0.434	0.884	0.384	0.3	0.753	0.196
	PHN	0.538	0.926	0.48	0.343	0.813	0.252
	**DA-HGL**	**0.575**	**0.931**	**0.572**	**0.401**	**0.864**	**0.353**
	exp2GO	0.496	0.844	0.43	0.363	0.728	0.251
	GrAPFI	0.225	0.594	0.085	0.158	0.624	0.036
	Domain-PFP	0.533	0.905	0.47	0.393	0.897	0.307
	DeepPFP	0.420	0.893	0.357	0.295	0.823	0.207

For Human proteins in the BP category, DA-HGL achieved an *F_max_* of 0.384, significantly outperforming Zhang (0.162) and Domain-PFP (0.310). Similar trends were observed in the MF and CC categories, where the *F_max_* values of DA-HGL, which are 0.379 and 0.401, surpassed those of Domain-PFP that are 0.366 and 0.393 respectively. Notably, AUC and AUPR of DA-HGL for Human BP annotations were 0.872 and 0.333, respectively, which are substantially higher than Domain-PFP’s 0.825 and 0.227. This highlights robustness of DA-HGL in distinguishing true positives from false positives in sparse annotation settings. In the case of yeast proteins, the performance advantages of DA-HGL were even more pronounced. For BP annotations, DA-HGL attained an *F_max_* of 0.515, compared to the values of 0.494 for Domain-PFP and 0.211 for Zhang. In yeast CC annotations, DA-HGL achieved an AUC of 0.572, far exceeding those of Domain-PFP and Zhang, which are 0.470 and 0.205. Yeast’s dense PPI network (high clustering coefficient, Table 3) facilitated domain-augmented propagation, while DA-HGL’s sequence embeddings mitigated sparsity in Human networks. LOOCV validated DA-HGL’s robustness in sparse annotation scenarios, driven by its integration of GO semantics and multimodal data.

**Table 3:** Topological comparison of yeast and human PPI networks

Features	Yeast	Human
Average number of neighbors	14.166	7.188
Characteristic path length	3.85	4.613
Clustering coefficient	0.363	0.26
Network density	0.005	0.001

### Ten-fold cross validation

To further validate DA-HGL’s robustness and generalizability, we performed 10-fold cross-validation (10-CV) on the CAFA3 benchmark dataset, partitioning proteins into 10 subsets for iterative training/testing. The results, as shown in Table 4, confirm the superior performance of DA-HGL across all metrics and ontology categories.

**Table 4:** Evaluation of different methods using 10-fold cross validation

Categories	Methods	Yeast			Human		
		*F_max_*	AUC	AUPR	*F_max_*	AUC	AUPR
BP	zhang	0.214	0.873	0.128	0.163	0.796	0.089
	DSCP	0.287	0.913	0.188	0.222	0.857	0.146
	NC	0.349	0.879	0.253	0.297	0.757	0.197
	PHN	0.454	0.92	0.387	0.309	0.827	0.202
	**DA-HGL**	**0.501**	**0.936**	**0.487**	**0.362**	**0.865**	**0.312**
	exp2GO	0.381	0.765	0.277	0.271	0.685	0.175
	GrAPFI	0.167	0.578	0.047	0.103	0.655	0.023
	Domain-PFP	0.426	0.825	0.326	0.291	0.797	0.199
	DeepPFP	0.344	0.850	0.258	0.276	0.817	0.195
MF	zhang	0.201	0.78	0.121	0.114	0.743	0.051
	DSCP	0.361	0.866	0.236	0.229	0.848	0.135
	NC	0.286	0.771	0.18	0.166	0.692	0.071
	PHN	0.375	0.832	0.3	0.189	0.787	0.103
	**DA-HGL**	**0.529**	**0.893**	**0.513**	**0.369**	**0.873**	**0.295**
	exp2GO	0.346	0.679	0.232	0.201	0.63	0.093
	GrAPFI	0.358	0.645	0.202	0.278	0.7	0.108
	Domain-PFP	0.371	0.838	0.31	0.352	0.876	0.237
	DeepPFP	0.487	0.869	0.451	0.321	0.834	0.221
CC	zhang	0.281	0.847	0.208	0.164	0.756	0.087
	DSCP	0.343	0.902	0.276	0.252	0.835	0.151
	NC	0.434	0.876	0.374	0.3	0.742	0.188
	PHN	0.524	0.924	0.472	0.337	0.809	0.239
	**DA-HGL**	**0.571**	**0.93**	**0.562**	**0.385**	**0.858**	**0.33**
	exp2GO	0.425	0.752	0.321	0.299	0.661	0.185
	GrAPFI	0.212	0.586	0.08	0.157	0.619	0.036
	Domain-PFP	0.474	0.85	0.378	0.332	0.854	0.239
	DeepPFP	0.404	0.887	0.339	0.289	0.818	0.202

For Human proteins in the BP category, DA-HGL achieved an *F_max_* of 0.362, outperforming Zhang (0.163) and Domain-PFP (0.291). Similarly, in the MF and CC categories, the *F_max_* of DA-HGL, which are 0.369 and 0.385, surpassed those of Domain-PFP that are 0.352 and 0.332 respectively. In the case of yeast proteins, the performance advantages of DA-HGL were even more pronounced. For BP annotations, DA-HGL attained an *F_max_* of 0.501, compared to the values of 0.426 for Domain-PFP and 0.214 for Zhang. In yeast CC annotations, DA-HGL achieved an AUC of 0.93 and AUPR of 0.562, significantly outperforming Domain-PFP’s 0.85 and 0.378. Additionally, as shown in Table 2 and 4, DeepPFP performs strongly in MF but lags in BP and CC. This pattern aligns with expectations: Sequence-driven models like DeepPFP excel at capturing localized structural motifs governing MF but fundamentally lack mechanisms to model the cross-protein functional associations required for BP and CC prediction.

**Figure 2:**
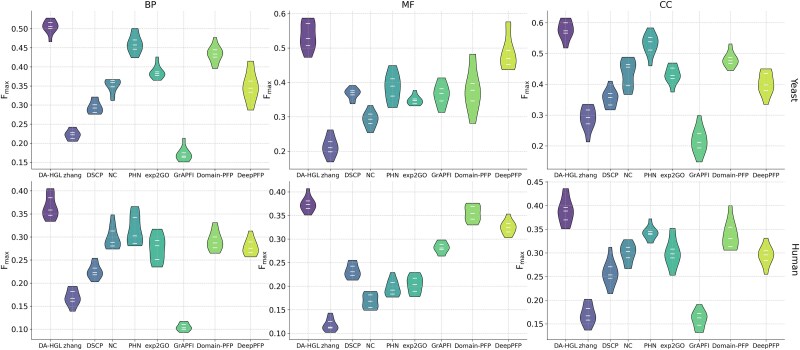
Comparative distribution of *F_max_* in yeast and human species. Violin plots depict the *F_max_* score distributions of DA-HGL and competing methods across BP, MF, and CC categories in yeast (top) and human (bottom). The width of each violin reflects data density, with DA-HGL (purple) showing narrower distributions (lower variance) and higher median values compared to other methods. Boxplots inside violins indicate quartiles (25th, 50th, and 75th percentiles) and outliers.

To evaluate stability, we analyzed *F_max_* distributions across 10-fold cross-validation rounds (Table 5, Fig. 2). DA-HGL consistently outperformed competitors in yeast, with *F_max_* concentrated in 0.45–0.55 (BP) and similar trends in MF/CC. In Human, DA-HGL maintained superior performance despite narrower margins. Its narrower *F_max_* distributions (versus scattered baselines like GrAPFI) indicated robustness to dataset variations, underscoring adaptability across species, and functional categories.

**Table 5:** Descriptive statistics of *F_max_* scores (mean ± standard deviation) for different methods

Methods	Yeast			Human		
	BP	MF	CC	BP	MF	CC
**DA-HGL**	**0.505 ± 0.018**	**0.535 ± 0.0391**	**0.577 ± 0.03**	**0.365 ± 0.025**	**0.374 ± 0.016**	**0.389 ± 0.027**
zhang	0.222 ± 0.013	0.213 ± 0.028	0.290 ± 0.037	0.169 ± 0.017	0.118 ± 0.013	0.169 ± 0.021
DSCP	0.294 ± 0.016	0.367 ± 0.016	0.355 ± 0.029	0.226 ± 0.016	0.232 ± 0.015	0.258 ± 0.026
NC	0.351 ± 0.019	0.292 ± 0.023	0.435 ± 0.043	0.297 ± 0.024	0.169 ± 0.015	0.300 ± 0.020
PHN	0.461 ± 0.024	0.386 ± 0.040	0.531 ± 0.034	0.314 ± 0.032	0.196 ± 0.017	0.342 ± 0.014
exp2GO	0.171 ± 0.018	0.365 ± 0.031	0.217 ± 0.042	0.104 ± 0.008	0.282 ± 0.011	0.160 ± 0.018
GrAPFI	0.386 ± 0.017	0.348 ± 0.013	0.430 ± 0.028	0.276 ± 0.028	0.204 ± 0.018	0.301 ± 0.029
Domain-PFP	0.434 ± 0.022	0.375 ± 0.060	0.480 ± 0.025	0.292 ± 0.020	0.355 ± 0.017	0.337 ± 0.030
DeepPFP	0.349 ± 0.039	0.486 ± 0.050	0.403 ± 0.037	0.278 ± 0.017	0.325 ± 0.014	0.295 ± 0.020

Statistical significance was validated via independent *t*-tests and Wilcoxon signed-rank tests [29] (Supplementary Tables 1 and 2). DA-HGL exhibited significantly higher *F_max_* means than all competitors (*P <* .05, Cohen’s d > 0.8), with extreme effect sizes (e.g. *d* = 19.29 for yeast BP). Nonparametric Wilcoxon tests confirmed robustness against non-normality (Shapiro–Wilk *P <* .05). After Benjamini-Hochberg FDR correction, all comparisons retained significance (e.g. corrected *P* = 3.68 × 10^−11^ for Zhang in yeast BP), demonstrating DA-HGL’s superiority across species and functional categories. With dual validation from *t*-test and Wilcoxon test, and robust results after FDR correction, DA-HGL exhibits reliability and generality as a gene function prediction tool, particularly suitable for large-scale biological dataset analysis.

### Functional prediction for disease-associated proteins

**Figure 3:**
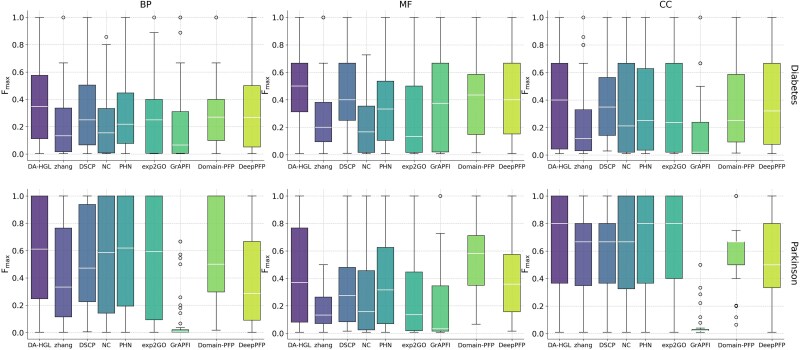
Performance comparison in disease-specific protein function prediction. Box plots show *F_max_* distributions of DA-HGL (purple) and eight baseline methods across BP, MF, and CC categories for diabetes (top) and Parkinson’s disease (bottom)-related proteins. Central line: median; box: interquartile range (25th–75th percentiles); whiskers: 1.5× interquartile range. DA-HGL exhibits higher medians and narrower distributions, indicating superior accuracy and stability. Outliers are shown as individual points.

DA-HGL demonstrated strong disease-specific utility, achieving significant *F_max_* improvements over Domain-PFP in diabetes (BP: +28.7%, MF: +26.4%, CC: +13.7%) and Parkinson’s disease (CC: +9.4%) (Supplementary Tables 3, Fig. 3). Statistical tests (Supplementary Tables 4 and 5) confirmed DA-HGL’s superiority (Cohen’s d > 0.8, FDR-corrected *P <* 0.05), with Domain-PFP showing negative effects in Parkinson’s MF (*d* = −0.41, Supplementary Table 4), likely due to its static domain modeling versus DA-HGL’s dynamic embeddings. Key GO terms aligned with disease mechanisms (e.g. “insulin receptor binding” for diabetes and “ubiquitin-dependent catabolism” for Parkinson’s) highlighting DA-HGL’s ability to uncover functional landscapes and therapeutic targets.

To further evaluate model performance and biological relevance in specific diseases, we analyzed Parkinson’s disease-associated PARK7/DJ-1 (UniProt: Q99497), whose loss-of-function mutations cause early-onset autosomal recessive Parkinson’s [30]. Focusing on BP (given weaker relevance of MF/CC to core pathology), we normalized cross-method BP predictions for Q99497 using Bayesian inference (sum probabilities = 1) and selected candidates with cumulative *P* ≤ .8 [11] (full annotations: Supplementary Table 6). DA-HGL outperformed Domain-PFP and DeepPFP in BP prediction for Q99497 (Precision = 0.108, Recall = 0.75, F1-score = 0.189 versus 0.058/0.292/0.097 and 0.118/0.083/0.097, respectively).

**Figure 4:**
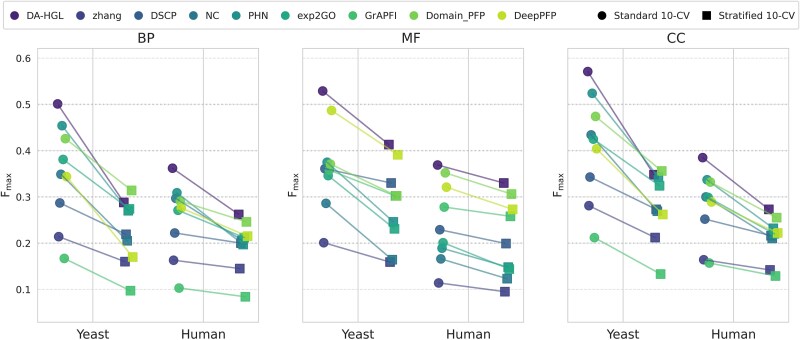
Visualization of GO semantic subnetwork and prediction results for PARK7/DJ-1 (Q99497). Nodes represent GO terms, edges denote “is_a”/“part_of” relationships. Red nodes indicate GO terms uniquely predicted by DA-HGL (exemplified by GO:0034599, “cellular response to oxidative stress”). Yellow nodes indicate GO terms uniquely predicted by domain-PFP. Green nodes indicate GO terms uniquely predicted by DeepPFP. Blue nodes indicate GO terms predicted by DA-HGL and at least one of the baseline methods. Gray nodes represent the hierarchical path and were not predicted by any method.

Fig. 4 visualizes the Q99497-related GO-GO semantic subnetwork and predictions from the three methods. All methods predicted “negative regulation of apoptotic process” (GO:0043066, blue node), a term consistent with dopaminergic neuron loss in Parkinson’s disease [31]. DA-HGL and Domain-PFP both predicted “protein stabilization” (GO:0050821, blue node), aligning with PARK7’s role in mitochondrial homeostasis, a core disease mechanism [32]. Critically, only DA-HGL predicted “cellular response to oxidative stress” (GO:0034599, red node). The loss of this term compromises the antioxidant/sensor activity of PARK7/DJ-1 and consequently increases the vulnerability of dopaminergic neurons to oxidative damage, which is a pivotal step in pathogenesis [33].

DA-HGL’s predictions show stronger biological relevance and mechanistic explanatory power with Parkinson’s disease pathology. It successfully identified PARK7’s disease-relevant core functions (especially the response to oxidative stress), enabled by its dynamic domain embedding (capturing tissue/condition-specific functional contexts) and heterogeneous graph learning.

### Robustness analysis

To systematically verify the model advantages, generalization ability, and cold-start performance of DA-HGL, we designed three types of experiments: verification of the contribution of model components, grouped verification of homologous proteins, and verification of cold-start scenarios.

Validating the effectiveness and importance of each component in our model, we designed three targeted ablation experiments as follows:

(1) Without Sequence: The protein–protein similarity graph was constructed exclusively using domain architecture information (setting γ = 0 in Equation 7), while retaining the full heterogeneous graph learning module.

(2) Without Domain: Only sequence features were used to construct the similarity graph (γ = 1 in Equation 7), preserving the graph learning framework.

(3) Without Heterogeneous Graph Learning: While integrating both sequence and domain features through Equation 7, the core NMF optimization with dual Laplacian regularization (Equations 10–16) was replaced with the neighbour counting method NC [4].

As quantified in Table 6, systematic removal of key components revealed distinct functional dependencies across GO categories. Most critically, removing domain architectures caused a 52.3% drop in Human performance (0.369 → 0.176), supporting the principle that enzymatic activities are determined by domain combinations. Conversely, CC prediction is sequence-driven: yeast *F_max_* falls 22.1% (0.571 → 0.445) when sequences are removed, confirming the conservation of localization signals. Replacing the heterogeneous graph learner with the NC baseline damages all ontologies (yeast BP drops 24.9%), showing dual Laplacian regularization (τ*_P_*, τ*_G_*) is indispensable. Thus, DA-HGL operates through component synergy: sequence features provide localization blueprints, dynamic domain embeddings resolve functional contexts, and graph learning orchestrates multimodal integration while preserving ontological hierarchies.

**Table 6:** Ablation experiment results of DA-HGL. The best results are emphasized in bold

Categories	Methods	Yeast			Human		
		*F_max_*	AUC	AUPR	*F_max_*	AUC	AUPR
BP	Without sequence	0.399	0.922	0.352	0.304	0.859	0.240
	Without domain	0.452	0.870	0.380	0.268	0.721	0.159
	Without heterogeneous graph learning	0.376	0.93	0.321	0.292	0.871	0.202
	**DA-HGL**	**0.501**	**0.936**	**0.487**	**0.362**	**0.865**	**0.312**
MF	Without sequence	0.501	0.882	0.477	0.358	0.868	0.281
	Without domain	0.353	0.766	0.256	0.176	0.680	0.077
	Without heterogeneous graph learning	0.453	0.884	0.374	0.287	0.866	0.193
	**DA-HGL**	**0.529**	**0.893**	**0.513**	**0.369**	**0.873**	**0.295**
CC	Without sequence	0.445	0.909	0.405	0.316	0.846	0.247
	Without domain	0.541	0.865	0.470	0.309	0.718	0.191
	Without heterogeneous graph learning	0.439	0.922	0.424	0.305	0.854	0.248
	**DA-HGL**	**0.571**	**0.93**	**0.562**	**0.385**	**0.858**	**0.33**

**Figure 5:**
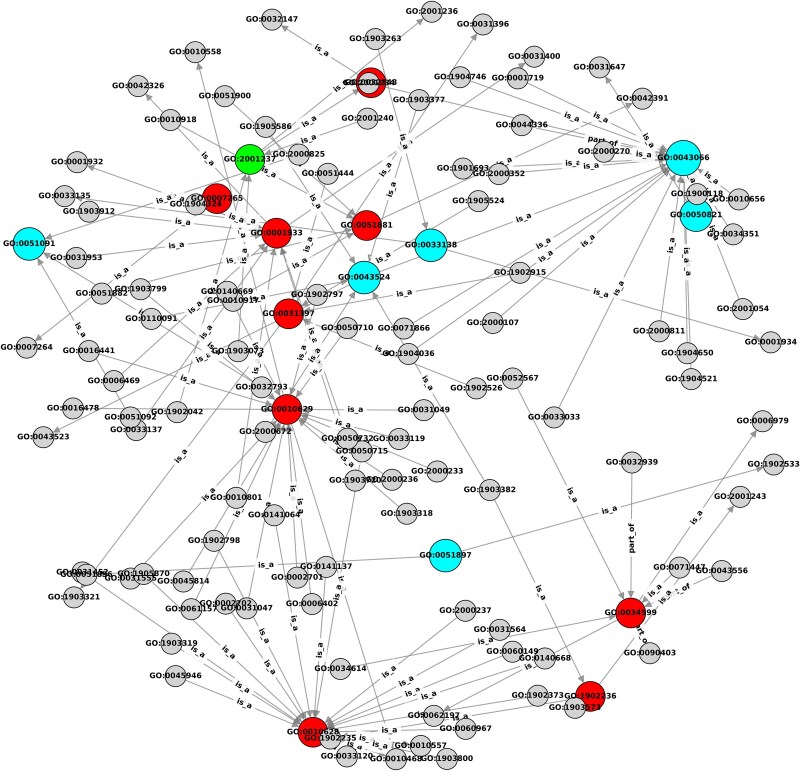
Performance under homology-aware versus standard 10-CV. Comparison of *F_max_* for yeast and human proteins across BP, MF, and CC ontologies is shown. Proteins are clustered by sequence similarity (>30% identity) to simulate novel protein annotation.

To rigorously evaluate potential performance overestimation arising from homologous protein contamination, we implemented a stratified 10-fold cross-validation strategy with homology-aware partitioning. Protein sequences were clustered hierarchically based on PseAAC similarity using a 30% sequence identity threshold, ensuring all homologs (>30% identity) resided within the same fold. This design simulates real-world scenarios for annotating novel proteins lacking close homologs in existing databases. As demonstrated in Fig. 5, performance degradation patterns across methods revealed critical insights into their dependency on sequence homology. Domain-based and sequence-driven approaches (Zhang: Δ*F_max_* = −0.054 in yeast BP; DSCP: −0.068; DeepPFP: −0.174) exhibited significant sensitivity due to restricted similarity metrics under homology exclusion. In contrast, topology-based methods like NC (Δ*F_max_* = −0.090) showed lower vulnerability, as PPI networks retain functional links beyond sequence homology. DA-HGL demonstrated moderate degradation (−0.039 in Human MF), attributable to its partial reliance on sequence features. Even so, DA-HGL maintained significant advantages under homology-restricted conditions. For example, in Human BP prediction, DA-HGL achieved an *F_max_* of 0.262, outperforming Domain-PFP (0.246) by 6.5% and DeepPFP (0.215) by 21.9%. Similarly, for yeast MF annotations, DA-HGL (0.413) outperformed Domain-PFP (0.302) by 36.8% and NC (0.164) by 151.8%. This resilience stems from its dynamic domain embeddings, trained via joint sequence-GO co-optimization, which resolves functional contexts through heterogeneous graph propagation. Such capability validates DA-HGL’s applicability to de novo protein annotation, particularly for orphan genes or taxon-specific innovations where homology-based methods falter.

To evaluate the applicability of DA-HGL in scenarios with missing input information, two cold-start experiments were designed: removing the domain information of proteins in the test set to simulate extreme scenarios of domain annotation absence, and removing the sequence features of test set proteins to assess predictive capability relying solely on nonsequence data. Tables 7 and 8 present the performance results of DA-HGL and corresponding comparison methods after removing domain information or sequence features of proteins in the test set, respectively. Domain-dependent methods such as GrAPFI and Domain-PFP exhibited severe performance degradation with a drop exceeding 77%. GrAPFI achieved an *F_max_* of 0, which is because such methods construct similarity networks based on domains; after removal, proteins in the test set become isolated nodes, losing the ability to transmit information. On the other hand, it is evident that DA-HGL significantly outperformed other competing methods: in the yeast BP task (*F_max_* = 0.475), it surpassed the best baseline PHN (0.301) by 57.8%, highlighting its ability to mitigate domain absence by integrating GO semantics and sequence features (PseAAC). The sequence-dependent method DeepPFP suffered a performance collapse, with an *F_max_* of only 0.034 in yeast BP, while DA-HGL remained competitive (yeast CC *F_max_* = 0.414). This is attributed to the fact that heterogeneous graph learning enables cross-modal inference through domain-function coherence (τ*_P_*) and GO hierarchical constraints (τ*_G_*). By leveraging dynamic domain embeddings and multisource data fusion, DA-HGL maintains predictive robustness in cold-start scenarios, providing a viable solution for functional annotation of novel proteins without prior knowledge.

**Table 7:** Performance comparison under domain removal cold-start scenario versus standard evaluation

Categories	Methods	Yeast			Human		
		Standard 10-CV	Domain removed	Reduction (%)	Standard 10-CV	Domain removed	Reduction (%)
BP	zhang	0.214	0.208	−2.8%	0.163	0.162	−0.6%
	DSCP	0.287	0.215	−25.1%	0.222	0.16	−27.9%
	PHN	0.454	0.301	−33.7%	0.309	0.224	−27.5%
	**DA-HGL**	**0.501**	**0.475**	**−5.2%**	**0.362**	**0.331**	**−8.6%**
	GrAPFI	0.167	0	−100.0%	0.103	0	−100.0%
	Domain-PFP	0.426	0.031	−92.7%	0.291	0.032	−89.0%
MF	zhang	0.201	0.195	−3.0%	0.114	0.111	−2.6%
	DSCP	0.361	0.197	−45.4%	0.229	0.116	−49.3%
	PHN	0.375	0.298	−20.5%	0.189	0.166	−12.2%
	**DA-HGL**	**0.529**	**0.389**	**−26.5%**	**0.369**	**0.22**	**−40.4%**
	GrAPFI	0.358	0	−100.0%	0.278	0	−100.0%
	Domain-PFP	0.371	0.085	−77.1%	0.352	0.066	−81.3%
CC	zhang	0.281	0.282	+0.4%	0.164	0.16	−2.4%
	DSCP	0.343	0.273	−20.4%	0.252	0.167	−33.7%
	PHN	0.524	0.405	−22.7%	0.337	0.276	−18.1%
	**DA-HGL**	**0.571**	**0.555**	**−2.8%**	**0.385**	**0.351**	**−8.8%**
	GrAPFI	0.212	0	−100.0%	0.157	0	−100.0%
	Domain-PFP	0.474	0.056	−88.2%	0.332	0.067	−79.8%

**Table 8:** Performance comparison under sequence feature removal cold-start scenario

Categories	Methods	Yeast			Human		
		Standard 10-CV	Sequence removed	Reduction (%)	Standard 10-CV	Sequence removed	Reduction (%)
BP	**DA-HGL**	**0.501**	**0.374**	−25.4%	**0.362**	**0.29**	−19.9%
	DeepPFP	0.344	0.034	−90.1%	0.276	0.035	−87.3%
MF	**DA-HGL**	**0.529**	**0.473**	−10.6%	**0.369**	**0.354**	−4.1%
	DeepPFP	0.487	0.097	−80.1%	0.321	0.054	−83.2%
CC	**DA-HGL**	**0.571**	**0.414**	−27.5%	**0.385**	**0.297**	−22.9%
	DeepPFP	0.404	0.073	−81.9%	0.289	0.051	−82.4%

### Parameter analysis

**Figure 6:**
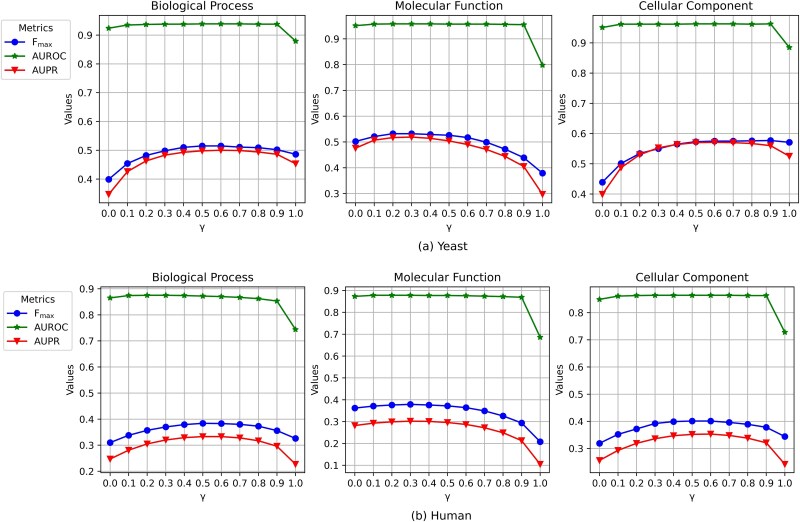
The analysis of parameters γ. The x-axis represents the weight parameter γ, which balances the contribution of sequence similarity versus domain architecture similarity in constructing the protein–protein similarity graph. The y-axis shows the corresponding *F_max_* score.

The balance between sequence and domain similarities, controlled by parameter γ (Equation 7), was optimized per functional category: γ = 0.5 (BP), 0.3 (MF), and 0.6 (CC) (Fig. 6). Higher γ for BP reflects dual dependencies on sequence conservation (e.g. interaction interfaces) and domain architectures (e.g. modular pathways). MF’s lower γ prioritizes domain-driven specificity (e.g. enzymatic motifs), while CC’s γ = 0.6 emphasizes sequence motifs for localization signals (e.g. nuclear targeting). This category-specific tuning aligns DA-HGL with biological principles, enhancing prediction accuracy across functional contexts.

### Evaluation on GO annotation 2024

In order to compare the performance of DA-HGL and other competing methods more comprehensively, we used another dataset i.e. derived from the GO Consortium [26] released on 8 September 2024. We filtered all proteins with experimental functional annotations with evidence codes EXP, IDA, IMP, IGI, IEP, TAS, and IC. Similarly, GO terms with annotations of less than 10 or more than 200 proteins were excluded, leaving 2913 and 6031 proteins on yeast and human, respectively. As shown in Table 9, DA-HGL still achieved the highest *F_max_* across all evaluation metrics and ontology categories. Notably, it excelled in MF prediction, where dynamic domain embeddings captured tissue-specific contexts (e.g. Yeast MF: *F_max_* = 0.541, +34.1% over DeepPFP). Additionally, DA-HGL’s performance drop from CAFA3 (Table 4) to GO-2024 was minimal (e.g. Yeast BP: −14.2% versus GrAPFI’s − 27.5%), demonstrating robustness to annotation shifts. This aligns with its design for sparse data via domain-GO co-optimization.

**Table 9:** Evaluation of different methods using 10-fold cross validation on GO annotation 2024.

Categories	Methods	Yeast			Human		
		*F_max_*	AUC	AUPR	*F_max_*	AUC	AUPR
BP	zhang	0.172	0.87	0.093	0.104	0.758	0.04
	DSCP	0.238	0.905	0.14	0.158	0.82	0.074
	NC	0.306	0.858	0.192	0.18	0.7	0.087
	PHN	0.387	0.911	0.3	0.196	0.782	0.097
	**DA-HGL**	**0.43**	**0.925**	**0.383**	**0.253**	**0.826**	**0.179**
	exp2GO	0.343	0.742	0.218	0.192	0.639	0.091
	GrAPFI	0.121	0.565	0.024	0.082	0.628	0.019
	Domain-PFP	0.317	0.801	0.238	0.209	0.76	0.11
	DeepPFP	0.277	0.826	0.184	0.185	0.776	0.09
MF	zhang	0.163	0.822	0.093	0.119	0.748	0.053
	DSCP	0.307	0.896	0.188	0.249	0.86	0.153
	NC	0.295	0.792	0.183	0.17	0.68	0.069
	PHN	0.412	0.871	0.324	0.206	0.789	0.123
	**DA-HGL**	**0.541**	**0.914**	**0.504**	**0.42**	**0.883**	**0.34**
	exp2GO	0.362	0.693	0.245	0.218	0.624	0.111
	GrAPFI	0.334	0.637	0.179	0.24	0.718	0.091
	Domain-PFP	0.317	0.865	0.257	0.357	0.896	0.25
	DeepPFP	0.401	0.835	0.323	0.321	0.828	0.225
CC	zhang	0.25	0.847	0.167	0.172	0.772	0.093
	DSCP	0.314	0.901	0.225	0.239	0.842	0.145
	NC	0.414	0.87	0.346	0.304	0.745	0.199
	PHN	0.511	0.921	0.45	0.345	0.816	0.248
	**DA-HGL**	**0.557**	**0.923**	**0.546**	**0.38**	**0.864**	**0.332**
	exp2GO	0.443	0.759	0.338	0.29	0.658	0.179
	GrAPFI	0.186	0.578	0.056	0.124	0.622	0.033
	Domain-PFP	0.478	0.855	0.364	0.287	0.848	0.21
	DeepPFP	0.385	0.869	0.292	0.256	0.79	0.161

## Discussion

DA-HGL’s superiority over sequence-driven models like DeepPFP is evident in its holistic data integration. While DeepPFP excels in MF prediction by capturing localized structural motifs (e.g. Yeast MF *F_max_* = 0.487), it underperformed in BP and CC categories due to its inability to model cross-protein functional associations. For example, DeepPFP failed to predict PARK7’s key BP term “cellular response to oxidative stress” (GO:0034599) in Parkinson’s disease, a mechanism that requires context-aware domain embeddings and GO hierarchy propagation.

DA-HGL’s dynamic domain embeddings enable tissue-specific predictions, as demonstrated in the PARK7/DJ-1 case (Fig. 5). Unlike Domain-PFP and DeepPFP, DA-HGL exclusively identified GO:0034599, aligning with PARK7’s role in mitigating neuronal oxidative stress—a core Parkinson’s mechanism. Similarly, for diabetes, DA-HGL predicted “insulin receptor binding” (GO:0005158) with 28.7% higher *F_max_* than baselines.

When domain or sequence features were removed (Tables 7 and 8), DA-HGL maintained competitive performance (e.g. Yeast BP *F_max_* = 0.475 without domains), while domain-dependent methods (GrAPFI, Domain-PFP) collapsed (*F_max_* → 0). This resilience stemmed from heterogeneous graph propagation and GO semantic constraints.

DA-HGL relies on GO annotation completeness, which may limit performance for understudied species. Integration of structural data (e.g. AlphaFold [34]) could enhance context-dependent predictions, especially for proteins with dynamic conformations. Applying DA-HGL to cancer proteomes may further validate its clinical utility.

**Key Points**

- DA-HGL learns context-aware domain embeddings via sequence-GO co-optimization, enabling tissue-specific predictions (e.g. neuronal oxidative stress response in Parkinson’s disease) for sparsely annotated proteins.
- DA-HGL constructs a weighted GO-GO semantic graph using direct parent–child links and enforces hierarchical consistency through non-negative matrix factorization, resolving annotation inconsistencies.
- DA-HGL mitigates cold-start challenges (e.g. achieving *F_max_* = 0.475 in yeast BP without domain annotations) by integrating alignment-free sequence features and domain-augmented similarities.
- DA-HGL identifies disease-specific functional terms (e.g. “insulin receptor binding” (GO:0005158) in diabetes and “cellular response to oxidative stress” (GO:0034599) in Parkinson’s disease), revealing core pathological mechanisms and therapeutic targets.
- These biologically interpretable predictions stem from enforcing domain-function coherence and GO hierarchical constraints, ensuring alignment with disease mechanisms.

## Supplementary Material

Supplementary_Tables_bbaf511

## Data Availability

Data and code are available at https://github.com/husaiccsu/DA-HGL.
