# Supplementary material for: DA-HGL: a domain-augmented heterogeneous graph learning framework for protein function prediction
Source: Brief Bioinform. 2025 Sep 28;26(5):bbaf511. doi: 10.1093/bib/bbaf511 (PMC12476837; doi:10.1093/bib/bbaf511)
Supplement: Supplementary_Tables_bbaf511 [file supplementary_tables_bbaf511.docx]

Supplementary Information for

**DA-HGL: A Domain-Augmented Heterogeneous Graph Learning Framework for Protein Function Prediction**

**Sai Hu^1^, Wei Zhang^2,3^, Bihai Zhao^2,3*^**

^1^ School of Mathematics, Changsha University, Changsha, Hunan 410022, China

^2^ School of Computer Science and Engineering, Changsha University, Changsha, Hunan 410022, China

^3^ Hunan Provincial Key Laboratory of Industrial Internet Technology and Security, Changsha University, Changsha, Hunan 410022, China

Supplementary Table 1. Statistical Significance, Effect Size, and Mean Differences Between DA-HGL and Competing Methods

| Categories | Comparison | Yeast | | | Human | | |
| --- | --- | --- | --- | --- | --- | --- | --- |
|  |  | *p* | Cohen's d | Mean Difference | *p* | Cohen's d | Mean Difference |
| BP | zhang | 9.21E-12 | 19.2890 | 0.2828 | 1.92E-10 | 9.7182 | 0.196 |
|  | DSCP | 5.32E-11 | 13.1083 | 0.2107 | 5.51E-09 | 7.1421 | 0.1394 |
|  | NC | 2.11E-08 | 8.9402 | 0.1538 | 5.54E-08 | 2.9406 | 0.0678 |
|  | PHN | 0.00015 | 2.2103 | 0.0446 | 3.22E-06 | 1.8948 | 0.0512 |
|  | exp2GO | 1.03E-07 | 7.1649 | 0.1187 | 1.86E-08 | 3.5635 | 0.0895 |
|  | GrAPFI | 1.01E-12 | 19.8161 | 0.334 | 1.77E-10 | 15.0673 | 0.261 |
|  | Domain-PFP | 2.03E-06 | 3.7304 | 0.0713 | 6.67E-08 | 3.4354 | 0.0736 |
|  | DeepPFP | 5.74E-08 | 5.3883 | 0.1558 | 3.58E-06 | 4.3105 | 0.087 |
| MF | zhang | 1.07E-10 | 10.0761 | 0.3224 | 1.96E-11 | 18.8522 | 0.2557 |
|  | DSCP | 4.53E-08 | 5.996 | 0.168 | 2.85E-08 | 9.7289 | 0.1412 |
|  | NC | 1.73E-08 | 8.0614 | 0.243 | 2.49E-10 | 13.9959 | 0.205 |
|  | PHN | 5.17E-09 | 4.0151 | 0.1496 | 8.11E-10 | 11.5313 | 0.1772 |
|  | exp2GO | 2.70E-08 | 6.8209 | 0.1872 | 7.40E-09 | 10.6146 | 0.1698 |
|  | GrAPFI | 2.62E-08 | 5.1109 | 0.1704 | 1.45E-08 | 7.1241 | 0.0916 |
|  | Domain-PFP | 5.56E-05 | 3.348 | 0.1606 | 0.006638 | 1.237 | 0.019 |
|  | DeepPFP | 0.001537 | 1.1784 | 0.0498 | 6.40E-05 | 3.4126 | 0.0486 |
| CC | zhang | 2.36E-12 | 8.8769 | 0.2867 | 7.91E-10 | 9.7187 | 0.2196 |
|  | DSCP | 4.43E-11 | 7.8639 | 0.2216 | 1.17E-07 | 5.1924 | 0.1308 |
|  | NC | 3.37E-08 | 3.9865 | 0.1413 | 1.08E-07 | 3.9749 | 0.0888 |
|  | PHN | 0.000129 | 1.4936 | 0.0458 | 5.20E-06 | 2.2966 | 0.0464 |
|  | exp2GO | 9.44E-10 | 5.2897 | 0.1461 | 2.38E-06 | 3.3057 | 0.0882 |
|  | GrAPFI | 1.81E-11 | 10.3745 | 0.3599 | 2.23E-10 | 10.5305 | 0.2288 |
|  | Domain-PFP | 1.09E-07 | 3.6622 | 0.0966 | 4.07E-05 | 1.934 | 0.0519 |
|  | DeepPFP | 1.67E-07 | 5.4358 | 0.1739 | 1.99E-05 | 4.1689 | 0.0941 |

Supplementary Table 2. Wilcoxon Signed-Rank Test Results and FDR-Adjusted p-values for Performance Comparisons

| Categories | Comparison | Yeast | | | Human | | |
| --- | --- | --- | --- | --- | --- | --- | --- |
|  |  | Wilcoxon | t-test  (FDR) | Wilcoxon  (FDR) | Wilcoxon | t-test  (FDR) | Wilcoxon  (FDR) |
| BP | zhang | 0.001953 | 3.68E-11 | 0.001953 | 0.001953 | 7.70E-10 | 0.001953 |
|  | DSCP | 0.001953 | 1.42E-10 | 0.001953 | 0.001953 | 1.47E-08 | 0.001953 |
|  | NC | 0.001953 | 4.21E-08 | 0.001953 | 0.001953 | 8.86E-08 | 0.001953 |
|  | PHN | 0.001953 | 0.000154 | 0.001953 | 0.001953 | 3.58E-06 | 0.001953 |
|  | exp2GO | 0.001953 | 1.36E-07 | 0.001953 | 0.001953 | 3.71E-08 | 0.001953 |
|  | GrAPFI | 0.001953 | 8.18E-12 | 0.001953 | 0.001953 | 7.70E-10 | 0.001953 |
|  | Domain-PFP | 0.001953 | 2.32E-06 | 0.001953 | 0.001953 | 9.50E-08 | 0.001953 |
|  | DeepPFP | 0.001953 | 9.19E-08 | 0.001953 | 0.001953 | 3.58E-06 | 0.001953 |
| MF | zhang | 0.001953 | 8.59E-10 | 0.001953 | 0.001953 | 1.57E-10 | 0.002232 |
|  | DSCP | 0.001953 | 6.04E-08 | 0.001953 | 0.001953 | 3.80E-08 | 0.002232 |
|  | NC | 0.001953 | 4.32E-08 | 0.001953 | 0.001953 | 9.97E-10 | 0.002232 |
|  | PHN | 0.001953 | 2.07E-08 | 0.001953 | 0.001953 | 2.16E-09 | 0.002232 |
|  | exp2GO | 0.001953 | 4.32E-08 | 0.001953 | 0.001953 | 1.47E-08 | 0.002232 |
|  | GrAPFI | 0.001953 | 4.32E-08 | 0.001953 | 0.001953 | 2.28E-08 | 0.002232 |
|  | Domain-PFP | 0.001953 | 9.29E-05 | 0.001953 | 0.013672 | 0.006638 | 0.013672 |
|  | DeepPFP | 0.005859 | 0.001537 | 0.005859 | 0.001953 | 7.32E-05 | 0.002232 |
| CC | zhang | 0.001953 | 1.89E-11 | 0.001953 | 0.001953 | 3.16E-09 | 0.001953 |
|  | DSCP | 0.001953 | 1.18E-10 | 0.001953 | 0.001953 | 2.34E-07 | 0.001953 |
|  | NC | 0.001953 | 5.39E-08 | 0.001953 | 0.001953 | 2.34E-07 | 0.001953 |
|  | PHN | 0.001953 | 0.000129 | 0.001953 | 0.001953 | 6.93E-06 | 0.001953 |
|  | exp2GO | 0.001953 | 1.89E-09 | 0.001953 | 0.001953 | 3.81E-06 | 0.001953 |
|  | GrAPFI | 0.001953 | 7.23E-11 | 0.001953 | 0.001953 | 1.75E-09 | 0.001953 |
|  | Domain-PFP | 0.001953 | 7.00E-08 | 0.001953 | 0.001953 | 4.07E-05 | 0.001953 |
|  | DeepPFP | 0.001953 | 1.91E-07 | 0.001953 | 0.001953 | 2.28E-05 | 0.001953 |

Supplementary Table 3. Fmax scores (mean ± standard deviation) for disease-associated protein function prediction.

| Methods | Diabetes | | | Parkinson | | |
| --- | --- | --- | --- | --- | --- | --- |
|  | BP | MF | CC | BP | MF | CC |
| **DA-HGL** | **0.377±0.309** | **0.507±0.291** | **0.415±0.334** | **0.583±0.375** | **0.415±0.355** | **0.684±0.347** |
| zhang | 0.211±0.232 | 0.293±0.279 | 0.240±0.278 | 0.450±0.377 | 0.169±0.142 | 0.593±0.319 |
| DSCP | 0.314±0.276 | 0.479±0.313 | 0.393±0.289 | 0.498±0.357 | 0.388±0.365 | 0.612±0.304 |
| NC | 0.229±0.244 | 0.210±0.207 | 0.322±0.336 | 0.554±0.395 | 0.288±0.338 | 0.617±0.353 |
| PHN | 0.292±0.281 | 0.382±0.308 | 0.354±0.307 | 0.583±0.402 | 0.409±0.376 | 0.670±0.350 |
| exp2GO | 0.260±0.270 | 0.265±0.284 | 0.327±0.331 | 0.544±0.402 | 0.265±0.333 | 0.634±0.363 |
| GrAPFI | 0.185±0.250 | 0.374±0.337 | 0.159±0.244 | 0.088±0.187 | 0.243±0.354 | 0.070±0.125 |
| Domain-PFP | 0.293±0.230 | 0.401±0.263 | 0.365±0.312 | 0.600±0.343 | 0.546±0.301 | 0.625±0.229 |
| DeepPFP | 0.321±0.295 | 0.449±0.327 | 0.393±0.335 | 0.417±0.366 | 0.394±0.304 | 0.562±0.315 |

Supplementary Table 4. Statistical significance and effect sizes from T-tests for disease-associated protein predictions.

| Categories | Comparison | Diabetes | | | Parkinson | | |
| --- | --- | --- | --- | --- | --- | --- | --- |
|  |  | *p* | Cohen's d | Mean Difference | *p* | Cohen's d | Mean Difference |
| BP | zhang | 2.34E-05 | 0.6122 | 0.1660 | 0.0006 | 0.3583 | 0.1333 |
|  | DSCP | 0.0193 | 0.2148 | 0.0625 | 0.0136 | 0.2362 | 0.0855 |
|  | NC | 2.63E-06 | 0.5353 | 0.1479 | 0.2652 | 0.0767 | 0.0292 |
|  | PHN | 0.0137 | 0.2906 | 0.0851 | 0.9954 | 0.0005 | 0.0002 |
|  | exp2GO | 0.0002 | 0.4060 | 0.1170 | 0.2184 | 0.1019 | 0.0392 |
|  | GrAPFI | 1.61E-06 | 0.6878 | 0.1920 | 2.04E-09 | 1.6909 | 0.4956 |
|  | Domain-PFP | 0.0047 | 0.3120 | 0.0844 | 0.706 | -0.0473 | -0.0168 |
|  | DeepPFP | 0.1323 | 0.1872 | 0.0561 | 0.0068 | 0.4537 | 0.1663 |
| MF | zhang | 0.0002 | 0.7583 | 0.2138 | 0.0066 | 0.9338 | 0.2453 |
|  | DSCP | 0.3869 | 0.0920 | 0.0275 | 0.7589 | 0.0774 | 0.0271 |
|  | NC | 8.47E-09 | 1.1863 | 0.2965 | 0.2779 | 0.3763 | 0.1267 |
|  | PHN | 0.0087 | 0.4192 | 0.1242 | 0.9589 | 0.0155 | 0.0055 |
|  | exp2GO | 7.54E-07 | 0.8492 | 0.2415 | 0.2392 | 0.4485 | 0.1499 |
|  | GrAPFI | 0.0013 | 0.4254 | 0.1325 | 0.0058 | 0.4975 | 0.1714 |
|  | Domain-PFP | 0.0465 | 0.3830 | 0.1052 | 0.2452 | -0.4108 | -0.1314 |
|  | DeepPFP | 0.1432 | 0.1895 | 0.0581 | 0.6770 | 0.0647 | 0.0208 |
| CC | zhang | 9.41E-05 | 0.5791 | 0.1759 | 0.0008 | 0.2773 | 0.0913 |
|  | DSCP | 0.5373 | 0.0742 | 0.0229 | 0.0170 | 0.2241 | 0.0722 |
|  | NC | 0.0087 | 0.2818 | 0.0933 | 0.0179 | 0.1936 | 0.0669 |
|  | PHN | 0.1773 | 0.1936 | 0.0614 | 0.6280 | 0.0409 | 0.0141 |
|  | exp2GO | 0.0883 | 0.2699 | 0.0887 | 0.1333 | 0.1439 | 0.0504 |
|  | GrAPFI | 7.79E-06 | 0.8869 | 0.2566 | 3.1E-12 | 2.3897 | 0.6148 |
|  | Domain-PFP | 0.4330 | 0.1592 | 0.0509 | 0.3478 | 0.2039 | 0.0592 |
|  | DeepPFP | 0.6325 | 0.0667 | 0.0221 | 0.0344 | 0.3738 | 0.1223 |

Supplementary Table 5. Wilcoxon signed-rank test results and FDR-adjusted p-values for disease-associated protein predictions.

| Categories | Comparison | Diabetes | | | Parkinson | | |
| --- | --- | --- | --- | --- | --- | --- | --- |
|  |  | Wilcoxon | t-test  (FDR) | Wilcoxon  (FDR) | Wilcoxon | t-test  (FDR) | Wilcoxon  (FDR) |
| BP | zhang | 1.63E-05 | 6.25E-05 | 4.35E-05 | 0.00022 | 0.00258 | 0.00089 |
|  | DSCP | 0.02190 | 0.02203 | 0.02502 | 0.01043 | 0.02710 | 0.02086 |
|  | NC | 7.57E-08 | 1.05E-05 | 3.03E-07 | 0.04866 | 0.353584 | 0.07786 |
|  | PHN | 0.00021 | 0.01823 | 0.00034 | 0.16581 | 0.99545 | 0.18950 |
|  | exp2GO | 5.10E-05 | 0.00039 | 0.00010 | 0.16151 | 0.34952 | 0.18950 |
|  | GrAPFI | 2.77E-08 | 1.05E-05 | 2.22E-07 | 1.74E-07 | 1.63E-08 | 1.39E-06 |
|  | Domain-PFP | 0.00521 | 0.00750 | 0.00695 | 0.96718 | 0.80689 | 0.96718 |
|  | DeepPFP | 0.06529 | 0.13233 | 0.06529 | 0.00107 | 0.01803 | 0.00286 |
| MF | zhang | 0.00029 | 0.00047 | 0.00078 | 0.01477 | 0.02655 | 0.05909 |
|  | DSCP | 0.21497 | 0.38688 | 0.21497 | 0.67912 | 0.86731 | 0.67912 |
|  | NC | 1.33E-07 | 6.78E-08 | 1.06E-06 | 0.03313 | 0.44463 | 0.08835 |
|  | PHN | 0.00238 | 0.01386 | 0.0038 | 0.27737 | 0.95894 | 0.36983 |
|  | exp2GO | 1.84E-06 | 3.01E-06 | 7.37E-06 | 0.05508 | 0.44463 | 0.11015 |
|  | GrAPFI | 0.00063 | 0.00252 | 0.00125 | 0.00098 | 0.02655 | 0.00785 |
|  | Domain-PFP | 0.10666 | 0.06206 | 0.12189 | 0.12393 | 0.44463 | 0.19829 |
|  | DeepPFP | 0.03995 | 0.16363 | 0.05327 | 0.42653 | 0.86731 | 0.48746 |
| CC | zhang | 0.00011 | 0.00038 | 0.00043 | 0.00179 | 0.00317 | 0.00716 |
|  | DSCP | 0.47271 | 0.61410 | 0.54024 | 0.01231 | 0.03571 | 0.02461 |
|  | NC | 0.00179 | 0.02317 | 0.00477 | 0.01754 | 0.03571 | 0.02806 |
|  | PHN | 0.11843 | 0.28367 | 0.23686 | 0.02763 | 0.62805 | 0.03684 |
|  | exp2GO | 0.15136 | 0.17669 | 0.24218 | 0.13522 | 0.17777 | 0.13522 |
|  | GrAPFI | 9.29E-06 | 6.23E-05 | 7.43E-05 | 1.58E-07 | 2.48E-11 | 1.26E-06 |
|  | Domain-PFP | 0.42357 | 0.57731 | 0.54024 | 0.12843 | 0.39752 | 0.13522 |
|  | DeepPFP | 0.56143 | 0.63252 | 0.56143 | 0.00477 | 0.05502 | 0.01271 |

Supplementary Table 6. Manual GO Annotations for Q99497 (PARK7).

| Ontology | Accession | Name |
| --- | --- | --- |
| BP | GO:0032148 | activation of protein kinase B activity |
| BP | GO:0070301 | cellular response to hydrogen peroxide |
| BP | GO:0034599 | cellular response to oxidative stress |
| BP | GO:0043066 | negative regulation of apoptotic process |
| BP | GO:0060548 | obsolete negative regulation of cell death |
| BP | GO:1902236 | negative regulation of endoplasmic reticulum stress-induced intrinsic apoptotic signaling pathway |
| BP | GO:2001237 | negative regulation of extrinsic apoptotic signaling pathway |
| BP | GO:0010629 | negative regulation of gene expression |
| BP | GO:0043524 | negative regulation of neuron apoptotic process |
| BP | GO:1901215 | obsolete negative regulation of neuron death |
| BP | GO:0032435 | negative regulation of proteasomal ubiquitin-dependent protein catabolic process |
| BP | GO:0032091 | negative regulation of protein binding |
| BP | GO:0006469 | negative regulation of protein kinase activity |
| BP | GO:0001933 | negative regulation of protein phosphorylation |
| BP | GO:0031397 | negative regulation of protein ubiquitination |
| BP | GO:0010628 | positive regulation of gene expression |
| BP | GO:0032757 | positive regulation of interleukin-8 production |
| BP | GO:0033138 | positive regulation of peptidyl-serine phosphorylation |
| BP | GO:0051897 | positive regulation of phosphatidylinositol 3-kinase/protein kinase B signal transduction |
| BP | GO:0051091 | positive regulation of DNA-binding transcription factor activity |
| BP | GO:2000679 | positive regulation of transcription regulatory region DNA binding |
| BP | GO:0050821 | protein stabilization |
| BP | GO:0007265 | Ras protein signal transduction |
| BP | GO:0051881 | regulation of mitochondrial membrane potential |
| MF | GO:0050681 | nuclear androgen receptor binding |
| MF | GO:0005507 | copper ion binding |
| MF | GO:0019900 | kinase binding |
| MF | GO:0003729 | mRNA binding |
| MF | GO:0008233 | peptidase activity |
| MF | GO:0005102 | signaling receptor binding |
| MF | GO:0070491 | DNA-binding transcription factor binding |
| MF | GO:0097110 | scaffold protein binding |
| MF | GO:0003713 | transcription coactivator activity |
| MF | GO:0008134 | transcription factor binding |
| MF | GO:1990381 | ubiquitin-specific protease binding |
| CC | GO:0000785 | chromatin |
| CC | GO:0048471 | perinuclear region of cytoplasm |
| CC | GO:0016605 | PML body |

Note: Although the MF/CC functions have relatively weak direct relevance to the core pathological mechanisms of Parkinson's disease (such as neuronal loss and oxidative stress), this table lists all experimentally validated functions for the sake of data integrity. The case analysis focuses on BP functions, as they involve key disease-related biological processes including apoptotic regulation and oxidative stress response.
